# Integration of Artificial Intelligence Into Sociotechnical Work Systems—Effects of Artificial Intelligence Solutions in Medical Imaging on Clinical Efficiency: Protocol for a Systematic Literature Review

**DOI:** 10.2196/40485

**Published:** 2022-12-01

**Authors:** Katharina Wenderott, Nikoloz Gambashidze, Matthias Weigl

**Affiliations:** 1 Institute for Patient Safety University Hospital Bonn Bonn Germany

**Keywords:** artificial intelligence, clinical care, clinical efficiency, sociotechnical work system, sociotechnical, review methodology, systematic review, facilitator, barrier, diagnostic, diagnosis, diagnoses, digital health, adoption, implementation, literature review, literature search, search strategy, library science, medical librarian, narrative review, narrative synthesis

## Abstract

**Background:**

When introducing artificial intelligence (AI) into clinical care, one of the main objectives is to improve workflow efficiency because AI-based solutions are expected to take over or support routine tasks.

**Objective:**

This study sought to synthesize the current knowledge base on how the use of AI technologies for medical imaging affects efficiency and what facilitators or barriers moderating the impact of AI implementation have been reported.

**Methods:**

In this systematic literature review, comprehensive literature searches will be performed in relevant electronic databases, including PubMed/MEDLINE, Embase, PsycINFO, Web of Science, IEEE Xplore, and CENTRAL. Studies in English and German published from 2000 onwards will be included. The following inclusion criteria will be applied: empirical studies targeting the workflow integration or adoption of AI-based software in medical imaging used for diagnostic purposes in a health care setting. The efficiency outcomes of interest include workflow adaptation, time to complete tasks, and workload. Two reviewers will independently screen all retrieved records, full-text articles, and extract data. The study’s methodological quality will be appraised using suitable tools. The findings will be described qualitatively, and a meta-analysis will be performed, if possible. Furthermore, a narrative synthesis approach that focuses on work system factors affecting the integration of AI technologies reported in eligible studies will be adopted.

**Results:**

This review is anticipated to begin in September 2022 and will be completed in April 2023.

**Conclusions:**

This systematic review and synthesis aims to summarize the existing knowledge on efficiency improvements in medical imaging through the integration of AI into clinical workflows. Moreover, it will extract the facilitators and barriers of the AI implementation process in clinical care settings. Therefore, our findings have implications for future clinical implementation processes of AI-based solutions, with a particular focus on diagnostic procedures. This review is additionally expected to identify research gaps regarding the focus on seamless workflow integration of novel technologies in clinical settings.

**Trial Registration:**

PROSPERO CRD42022303439; https://www.crd.york.ac.uk/prospero/display_record.php?RecordID=303439

**International Registered Report Identifier (IRRID):**

PRR1-10.2196/40485

## Introduction

In medicine, vast changes in patient care because the development of artificial intelligence (AI) is foreseen and ongoing. AI is broadly defined as “the ability of computers to perform tasks that normally require human intelligence” [1]. The introduction of these technologies in medicine promises to improve the quality and safety in health care and accessibility of medical expertise [1]. In the future, AI-human collaboration can augment the ability of clinicians in health care delivery by extracting relevant information from big data sets or performing tasks with higher precision [2,3]. The areas where AI technologies can assist the health care professionals are manifold, for example, clinical diagnostics, decision-making, or health care administration [2,4,5]. These technologies “can be used as powerful tools and partners to enhance, extend, and expand human capabilities, delivering the types of care patients need, at the time and place they need them” [4].

When integrating AI applications into clinical practice, these technologies will become part of highly complex sociotechnical work systems. A model that considers the complexity and scope of the clinical care work environment is the systems engineering initiative for patient safety (SEIPS) 2.0 model [6]. On the basis of SEIPS 2.0, the conceptual model of workflow integration was developed to investigate the integration of a new technology into clinical work processes, which has also been applied to the integration of AI [7,8]. The model uses a sociotechnical system approach and proposes that the whole work system and workflow must be considered to evaluate the success of an AI technology implementation [8].

Some work systems in medicine are faster or more suitable in adopting AI-facilitated technologies. Especially, in specialties that are largely image-based or process big amounts of data, AI is expected to support physicians and improve patient care by leading to more effective and efficient diagnostics [9,10]. Health care providers in image-based medical disciplines handle a growing amount of imaging data that require thorough interpretation [11]. Moreover, the shortage of physicians in radiology and a limited time available per image to meet the current workload are common challenges [12]. The introduction of AI into clinical practice holds a significant potential for changes in clinicians’ duties and improvements such as advancing routine tasks and freeing clinicians’ time for other important tasks [1,2].

One of the main objectives in introducing AI into health care is efficiency improvement because AI is expected to take over not exceedingly complex but time-consuming tasks [1,13,14]. This goal can only be achieved if these technologies are seamlessly integrated into the existing clinical workflow [15]. Therefore, a correlation between workflow integration and usability outcomes, which include efficiency, effectiveness, and satisfaction, has been proposed [7,16]. Efficiency is defined as “resources used in relation to the results achieved. […] Typical resources include time, human effort, costs and materials” [16]. Drawing upon the conceptual model of workflow integration, efficiency-related clinician outcomes include the adaptation of workflow, time to complete tasks, and workload [7,13].

To our knowledge, there is currently no systematic literature review or structured synthesis available on whether the integration of AI into the clinical workflow is associated with improved efficiency. Therefore, comprehensive evidence is necessary, concerning the major promise of freeing physician time for other care activities, for example, direct patient care. As the potential fields of application for AI technologies in health care are diverse, we focus on AI used for medical imaging to enable comparability. In this review, efficiency-related clinician outcomes such as workflow adaptation, time to complete tasks, and workload will be considered. Moreover, reported facilitators or barriers for the successful integration of AI into the workflow will be reviewed as “workflow integration is crucial for making this kind of software [computer-aided detection based on AI] a success” [13].

Our systematic review addresses the following question: how do AI technologies influence the efficiency of workflows in medical imaging?

Specifically, it aims to synthesize the literature base concerning two specific objectives: (1) Identification and overall aggregation of the effects of AI technology implementation on efficiency-related clinician outcomes such as workflow adaptation, time to complete tasks, and clinicians’ workload; and (2) Description of the facilitators and barriers for the integration of AI into the workflow of medical imaging.

## Methods

### Protocol Registration and Reporting Information

A systematic literature review will be performed to assess the existing literature base and findings. The review’s protocol is registered in the PROSPERO database (registration: CRD42022303439). The protocol and subsequent systematic review follow the reporting guidelines of preferred reporting items for systematic review and meta-analysis protocols statement. The checklist is included in Multimedia Appendix 1.

### Eligibility Criteria and Study Design

Only original studies retrieved in full-text and published in peer-reviewed journals will be included. The review will include prospective observational and interventional studies such as randomized controlled trials and nonrandomized studies of interventions, for example, before–after studies and those with an interrupted time series design.

#### Population

We will include studies conducted in health care facilities such as hospitals, clinics, or outpatient settings using medical imaging. All types of health care professionals, including all age groups, sexes, professions, and qualifications, will be included from the hospital and clinical care settings.

#### Exposure and Intervention

Studies targeting AI used for medical imaging and its effects on health care professionals interacting with the technology will be eligible for inclusion in this review, including a broad range of AI solutions and clinical work settings. Regarding clinical medical imaging and diagnostics, AI can be defined as “any computer system that can correctly interpret health data, especially in its native form as observed by humans” [17]. AI is often used in this context to identify or forecast a disease state [17]. This review will exclusively focus on AI used for image data interpretation for diagnostic purposes as well as medical imaging [2]. Therefore, our working definition for AI used for medical imaging activities as well as clinical diagnostics in this study will be as follows: any computer system used to interpret imaging data to make a diagnosis, support an image-based clinical (intervention) task, or screen for a disease, a task previously reserved for specialists.

#### Comparators

Studies comparing the use of AI in clinical diagnostics and medical imaging with only human specialists will be the comparison of interest; however, it is not a necessary condition for studies to be included in this review.

#### Outcomes

##### Overview

**Figure 1:**
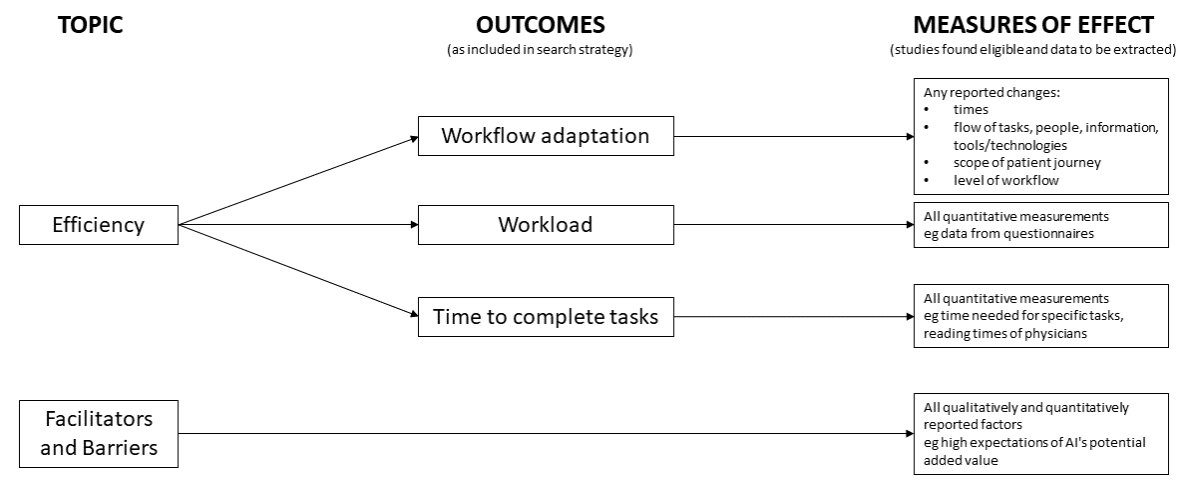
Outcomes and measures of effect in this review. AI: artificial intelligence.

Our central study objective is to investigate the impact of AI solutions for clinical diagnostics on the workflow efficiency in clinical care settings. On the basis of our theoretical background, we will focus on three associated outcomes, namely, (1) workflow adaptation, (2) workload, and (3) time-to-complete tasks. In addition, we will systematically assess any facilitators and barriers of AI integration into practice that are mentioned in eligible studies (Figure 1).

##### Workflow Adaptation

Workflow is defined as “the automation of a business process, in whole or part, during which documents, information or tasks are passed from one participant to another for action, according to a set of procedural rules” [18]. This definition was given by the workflow management coalition for business processes but can be also used for clinical contexts [18]. Thus, we will systematically evaluate the adaptation of the workflow in form of any reported changes to the existing processes due to the introduction of an AI technology.

##### Workload

Workload is defined as “the task demand of accomplishing mission requirements for the human operator” [19,20]. Measuring and analyzing clinical workload is “dependent on the tasks performed, the total time needed to complete the tasks and other care delivery needs of patients” [20,21]. Workload can be measured using objective measures, for example, cases seen or physiological data, and subjective measures such as questionnaires [22]. We will include all forms of quantitative workload measurements that compare the use of an AI software to traditional or previous methods such as pre-existing IT solutions, tools, and technologies in the workplace.

##### Time to Complete Tasks

New technologies provide opportunities to reduce the time needed to complete tasks, such as the time needed to examine magnetic resonance (MR) or computed tomography images [7,13]. Therefore, we will consider all reported measures on the time-to-task completion or duration. Time to complete tasks will be included if time changes on tasks of interest, such as diagnostic reading of MR images or writing of patient reports, are reported quantitatively with a comparison between the use of AI and traditional methods.

##### Facilitators and Barriers

A facilitator is defined as any factor that promotes or expands the integration or use of the AI system in the workflow. A barrier is defined as any factor that limits or restricts the integration or use of the AI system. The definitions were developed based on a systematic review by Niezen and Mathijssen [23], and the reported results will be classified according to these definitions. We will extract and synthesize facilitators and barriers in a narrative form using the Nonadoption, Abandonment, Scale-Up, Spread, and Sustainability framework for novel medical technologies in health care organizations [24].

#### Publication Types

We will include studies published from January 2000 onward because deep learning was developed in the early 2000s, which is thus marked as the beginning of a new area of AI use in medicine [25]. The article must be in English or German to be eligible for this review.

Owing to our rigorous scope, we limit our review to peer-reviewed journal articles and exclude dissertations, theses, and conference proceedings; as for the latter, the peer-review standards differ across conferences or disciplines. Furthermore, research on AI in medicine not used for medical imaging or diagnostics or research excluding the effects on the work system, such as studies on human interaction with the technology, will not be considered in this review.

### Search Strategy

Literature will be retrieved through a structured literature search in several electronic databases: MEDLINE (PubMed), Embase, PsycINFO, Web of Science, IEEE Xplore, and Cochrane Central Register of Controlled Trials. We will use further the snowball method to identify literature not detected through electronic databases, thus screening through the references of identified studies and using Google Scholar. Table 1 outlines the search strategy, following the PICO framework. Because we have decided that comparator is not a necessary condition to be included in this review, we did not list it in the search strategy (see eligibility criteria above). To expand the list of search terms, a preliminary search will be performed before the main search.

**Table 1:** Search strategy according to the PICO framework.

Classification	Connector	Search term
Population	—^a^	“hospital” OR “clinic” OR “healthcare” OR “health care delivery” OR “clinical care” OR “medical” OR physician* OR clinician* OR doctor* OR nurse* OR “health care professional” OR “patient care” OR patient* OR surg* OR “oncology” OR “radiology” OR “health information”
Intervention	AND	“artificial intelligence” OR “machine intelligence” OR “machine learning” OR “deep learning” OR “neural network” OR “natural language processing” OR “AI ” OR “automated image recognition” OR “decision-support” OR “AI application*”
Intervention	AND	“adoption” OR “deploy*” OR “implementation” OR “integration”
Intervention	AND	diagnos* OR “Magnetic Resonance Imaging” OR MRI OR “computer tomography” OR imag* OR detect* OR “data interpretation” OR “information system*” OR “health information technology*” OR “health IT*” OR “medical informatics” OR “electronic health record*” OR “medical record*” OR “patient data”
Outcomes	AND	“workload” OR “work reduction” OR load* OR “cognitive load” OR demand* OR time* OR stress* OR “satisfaction” OR “usability” OR “workflow” OR efficienc* OR “work system” OR “work adaptation” OR “turnaround” OR “clinician outcome” OR “performance”

^a^None.

### Screening and Selection Procedure

All retrieved articles will be imported into the software *Zotero*, an open-source reference management software [26]. For title and abstract screening, *Rayyan*, a web application for an initial title and abstract screening, will be used [27,28]. In the first step, the titles and abstracts will be independently screened by 2 reviewers who will undergo training to increase interrater agreement. In case of disagreement, a third researcher from the team will be consulted to solve the conflict in a discussion. If the disagreement cannot be solved through obtaining consensus, the 3 researchers will solve the conflict democratically, that is, majority vote. In the second step, full texts of all eligible publications will be retrieved. These will also be screened by 2 reviewers, and potential conflicts on whether the articles should be included will be resolved in a discussion moderated by a third member of the study team. Studies that are excluded in the process will be recorded. A flow diagram presenting the study selection process will be prepared, following the PRISMA (Preferred Reporting Items for Systematic Reviews and Meta-Analyses) 2020 flow diagram for new systematic reviews, which included searches of databases, registers, and other sources [29].

### Data Collection Procedure

The study data will be extracted by 1 author and imported into MS Excel (Microsoft Corp). The study data contain details on study characteristics, sample, setting, type of intervention, type and assessment of outcomes, statistical analyses, reported results, moderators or control of confounders, and further information of interest (Textbox 1). The studies and extracted data will be checked at random by another reviewer from the study team. To obtain an agreement on relevant data to be extracted, data from the first 5 studies will be extracted by both reviewers, and a guideline for data extraction will be developed. The extracted data will be divided into several main categories. If any information is missing, the authors of that particular study will be contacted for further details. In case of multiple publications on 1 study, only the key publication will be included.

**Main categories for data to be extracted.**

1. Study characteristics

- Authors
- Year of publication
- Location
- Study design

2. Sample

- Sample size
- Participants: demographics and professional characteristics

3. Setting

- Type of clinic
- Medical specialty
- Task

4. Type of intervention

- Artificial intelligence technology (category, reliability, and source)

5. Type and assessment of outcomes

- Workflow adaptation, workload, and times other reported outcome variables
- Facilitators and barriers (if reported)
- Sources of outcomes
- Assessment method (eg, interview, questionnaire, and observation)

6. Statistical analyses

- Types of statistical methods and analyses
- Means and variance metrics of outcomes (eg, standard deviations and confidence intervals)

7. Reported results

- Quantitative results
  - Coefficients (β, γ) and measures of strength of association between artificial intelligence and changes in outcome variables
  - Effect sizes (if reported or calculable)
  - *P* values
- Qualitative results
  - Named facilitators and barriers
  - Any reported analysis

8. Moderators or control of confounders

- Potential moderators or confounding variables (if reported)

9. Further information of potential interest

- Further information, for example, on limitations

### Study Appraisal and Risk of Bias (Quality) Assessment

To assess the methodological quality of the included studies, a standardized risk of bias assessment will be performed. Three established tools to assess the risk of bias, applied by two independent reviewers, will be used. Cochrane Risk of Bias Tool (Rob2) [30] will be used for randomized controlled trials. For nonrandomized studies, the risk of bias in nonrandomized studies of interventions tool [31] will be used. These tools address different sources of bias, including the steps from selection to reporting. For observational studies, a checklist of quality of reporting of observational longitudinal research [32] will be used. In case of disagreement, a third reviewer will be consulted until consensus is achieved.

### Strategy for Data Synthesis

First, we will qualitatively describe the overall sample and summarize the information extracted from each study. We will then provide an overview concerning the classification in our main categories (Textbox 1). The results of the risk of bias assessment will be provided in a narrative and tabular format. If an adequate set of studies of 5 or more studies is found eligible and the homogeneity level allows, we will perform a meta-analysis that reviews the effects of the introduction of AI on efficiency-associated outcomes. We will quantitatively synthesize data from the retrieved studies using the metafor package in R (R Core Team, R Foundation for Statistical Computing), which contains a set of functions for calculating meta-analyses such as effect-size calculation or model fitting to the data [33]. As we expect a level of heterogeneity of effects in the included studies, a random effects model will be used to estimate the average effect across studies. The heterogeneity across the included studies will be assessed using the Cochran Q test [34] and *I*^2^ statistic [35]. If the number of studies (at least 5 studies per group) and heterogeneity among them allow, subgroup analyses concerning specific characteristics within our eligibility criteria (ie, participants’ demographics, particular work settings, outcomes, study designs, and quality) will be performed.

If a meta-analysis is not possible, the results will be summarized in a narrative form and will also be presented in a tabular format. Regardless of the possibility of a meta-analysis, the results will be presented graphically to summarize the retrieved information in a user-friendly manner. We will also adopt a narrative synthesis approach for our additional outcomes, namely, facilitators and barriers. The narrative synthesis will be consistent with that of Strohm et al [36] who conducted an interview study on the factors facilitating and hindering the implementation of AI in radiology. They used the nonadoption, abandonment, scale-up, spread, and sustainability framework for new medical technologies in health care organizations, which will be also used in our data analysis [24,36].

### Meta-biases

Regarding the potential sources of meta-bias (eg, publication bias across studies and selective reporting) in the results of the review and meta-analysis, we plan to create a funnel plot, which plots study size against the reported effect size. If a publication bias occurs, the resulting scatterplot is asymmetric with more studies showing a positive than a negative result [37]. We will include at least 10 studies (if possible) to check for small-study effects [38-40]. Additionally, we will use the critical appraisal tool for systematic reviews on randomized or nonrandomized studies of health care interventions AMSTAR-2, which consists of 16 items assessing the quality of conduct of our systematic review [41].

### Confidence in Cumulative Evidence

The strength of the body of evidence will be assessed by using the Grading of Recommendations Assessment, Development and Evaluation, a system for rating the quality of evidence and strength of recommendations [42,43]. This rating system has been successfully used in clinical medicine, public health, and policy making, and more recently, in occupational and environmental health [44]. It supports the authors in rating their confidence whether the estimate of an effect is correct. In systematic reviews, the quality of evidence is rated separately for each outcome on a scale from high to very low [45].

## Results

The search and screening for the systematic literature review are anticipated to be finished in October 2022. Data extraction, quality appraisal, and subsequent data synthesis will begin in November 2022. The review is expected to be completed by April 2023, and the study results will be published in 2023.

## Discussion

### Principal Findings

We propose a protocol for a systematic review on the influence of AI technologies on workflow efficiency in clinical care settings. Our review will summarize the existing literature and provide a comprehensive overview on the work system effects of AI technologies in clinical care. This will focus on efficiency outcomes as these are promising factors in the integration of AI into clinical practice. To our knowledge, no systematic overview has been yet conducted on this subject.

The focus in our review will be on workflow and clinician outcomes in imaged-based disciplines as in these fields AI technologies are predominantly and continuously integrated into clinical care practice. Presumably, in the future, almost every medical specialty will interact with AI-based technologies because of a broad range of potential AI application fields in this domain [46]. Contrary to the popular belief that AI will replace radiologists or other health care staff, the future of medicine will rather depend on optimized interactions between AI and humans, enabling AI systems to augment the physician’s performance [12,47]. AI is foreseen to change clinicians’ work environments and affect their work processes such as task flow and workload [3,46]. Notwithstanding the various promises being proposed with the introduction of AI in real-world care environments, current evidence concerning its effects on clinicians’ workflow and practices is missing. Our review will therefore provide valuable insights into the existing evidence base on the immediate effects of AI implementation on work systems and clinician outcomes. Thus, our research synthesis will facilitate understanding if the current AI technologies live up to the expectation of significantly supporting clinicians in their work [48].

### Comparison to Previous Research

Notably, in light of the current gap between the broad utilization of AI for research purposes and few AI applications being applied in routine patient care, facilitating AI implementation and adoption into clinical care has become essential. Although academic publications on AI solutions for medical imaging, diagnostic, and therapeutic contexts are numerous, only a few real-world solutions have been yet officially approved and implemented in the health care sector [49]. Furthermore, we expect that only a fraction of these solutions has been systematically evaluated regarding their impact on clinician outcomes or workflow integration. This expectation is supported by the review of Asan and Choudhury [3] who demanded systematic research that addresses AI’s impact on clinical workflow and usability with emphasis on the importance of human factor research.

Because the seamless integration of AI is crucial for unfolding its potential in clinical practice, our review will specifically address the facilitators and barriers of implementation practices elicited from the retrieved studies [13]. The consideration of facilitating and hindering factors of AI adoption is an essential step in gaining a more detailed understanding of how AI implementation can be optimized in hospital and other clinical care settings. A study suggests that various process factors affect seamless AI adoption into hospital practices, such as a perceived high added value or hospital-wide innovation strategies, technical performance, and well-structured implementation processes [36]. We acknowledge that we will only extract the process characteristics from studies found eligible regarding clinician outcomes. Nevertheless, our synthesis approach, which draws upon a previously established framework, allows for a comprehensive understanding of AI implementation experiences and will expand the existing preliminary findings [36].

### Limitations

Our review will focus on AI used for medical imaging used for diagnostic purposes. AI applications offer a great potential for image-based specialties and address a pressing issue, namely, the vastly growing amount of imaging data that need thorough interpretation [15,47]. Significant technological advancements have been made recently through the development of AI solutions and their application into clinical practice [1,50,51]. We solely focus on this clinical domain and a specific clinical task (eg, image-based tasks and diagnostics) to strengthen internal and external validity as well as to allow comparability across the work settings included. Nonetheless, we capture a medical field with the most extensive availability of AI technologies already integrated into clinical routine practices.

The algorithms or features used in the AI technologies included in this review might be different; however, this is not of central interest for answering our research question. We will not assess the quality or clinical effectiveness of the AI systems because this is covered by numerous systematic reviews with regard to the specific task for which comparable AI solutions were developed, such as in the reviews by Kunze et al [52] or Chidambaram et al [53]. Therefore, no specific conclusions regarding the technologies or characteristics of AI will be drawn as we will focus solely on the work system effects.

To achieve our goal of summarizing the existing literature on the impact of AI implementation on clinician outcomes, we will establish a rigorous list of exclusion criteria regarding study design, setting, and population. Therefore, conclusions will only be drawn for the specific setting of work environments where AI is used for image-based and diagnostic purposes. We acknowledge that this may result in limited generalizability of our results. In future research, it would be valuable to compare the workflow integration of AI across different health care settings such as ambulant care settings or nursing facilities. Our review approach may be an exemplary approach on how to systematically aggregate research findings on AI workflow integration, which can be transferred to other health care sectors and clinical domains.

Our outcome variables of interest draw upon the conceptual model of workflow integration [7]. Our key focus will be on clinician outcomes, workflow, and efficiency—the key issues for AI introduction. Notably, we will only address clinician outcomes named in the model, namely, those related to workload and efficiency. For future research, it would be valuable to include further outcomes such as perceived use and acceptance. Furthermore, it would be interesting to augment research with concepts such as trust and technology characteristics as these are important determinants of AI adoption [36,54,55].

Regarding our key concepts extracted from the conceptual model of workflow integration [7], there is substantial heterogeneity of applicable terms in the literature; for example, time to complete tasks is a collective term for measures such as physician’s reading times [13] or time undertaken to review an image [56]. Moreover, some concepts used in this literature review, such as the use of AI in clinical diagnostics or facilitators and barriers for AI implementation, do not have a consistent definition in the literature. Therefore, we propose working definitions on the background of existing research [2,19,23]. Nonetheless, we acknowledge that key terms might be conceived differently in other contexts or publications. Thus, we limited the deviation from previous studies by conducting a pilot search and expanding our search terms to include common variants of key concepts.

### Conclusions

Our review and meta-analysis or systematic narrative data analysis will allow first systematic conclusions on how AI for medical and diagnostic imaging affects clinician efficiency outcomes. We expect to provide a structured overview and systematic synthesis of the current literature. Thus, the findings of our review are expected to expand the existing knowledge on how AI affects clinical efficiency in medical imaging. Particularly, by providing a quality appraisal of the included studies, we will identify shortcomings of the current research. Moreover, our review will help to recognize research gaps regarding the seamless workflow integration of novel technologies into clinical settings. Our findings will eventually also provide guidance on provider-centered design and application of AI-based solutions in clinical settings, with potential improvements in clinical safety and performance. Furthermore, our consideration of the facilitators and barriers of AI implementation will provide an evidence-based foundation for hospital leadership and practitioners to successfully manage AI implementation in patient care.

## Appendix

Multimedia Appendix 1 PRISMA-P Checklist.

## Abbreviations

AI: artificial intelligence
SEIPS: systems engineering initiative for patient safety
MR: magnetic resonance
PRISMA: Preferred Reporting Items for Systematic Reviews and Meta-Analyses
